# Efficient Patch-Wise Semantic Segmentation for Large-Scale Remote Sensing Images

**DOI:** 10.3390/s18103232

**Published:** 2018-09-25

**Authors:** Yan Liu, Qirui Ren, Jiahui Geng, Meng Ding, Jiangyun Li

**Affiliations:** 1 School of Automation & Electrical Engineering, University of Science and Technology Beijing, Beijing 100083, China; liuyan@ustb.edu.cn (Y.L.); s20160612@xs.ustb.edu.cn (Q.R.); g20168521@xs.ustb.edu.cn (J.G.); 2 Key Laboratory of Knowledge Automation for Industrial Processes, Ministry of Education, Beijing 100083, China; 3 Bayer HealthCare, Pittsburgh, PA 15238, USA; meng.ding@okstate.edu

**Keywords:** remote sensing, image segmentation, fully convolutional network, patch-wise, multi-scale

## Abstract

Efficient and accurate semantic segmentation is the key technique for automatic remote sensing image analysis. While there have been many segmentation methods based on traditional hand-craft feature extractors, it is still challenging to process high-resolution and large-scale remote sensing images. In this work, a novel patch-wise semantic segmentation method with a new training strategy based on fully convolutional networks is presented to segment common land resources. First, to handle the high-resolution image, the images are split as local patches and then a patch-wise network is built. Second, training data is preprocessed in several ways to meet the specific characteristics of remote sensing images, i.e., color imbalance, object rotation variations and lens distortion. Third, a multi-scale training strategy is developed to solve the severe scale variation problem. In addition, the impact of conditional random field (CRF) is studied to improve the precision. The proposed method was evaluated on a dataset collected from a capital city in West China with the Gaofen-2 satellite. The dataset contains ten common land resources (Grassland, Road, etc.). The experimental results show that the proposed algorithm achieves 54.96% in terms of mean intersection over union (*MIoU*) and outperforms other state-of-the-art methods in remote sensing image segmentation.

## 1. Introduction

Automatic remote sensing image analysis is an active research topic in the field of image processing, since its important applications in military, agriculture, environmental science, etc. Accurate image segmentation is a key technique in remote sensing to extract semantic information and achieve the automatic image analysis. There have been many existing valuable applications based on semantic segmentation already, such as urban classification [1], building extraction [2], aircraft recognition [3] and morphotectonic analysis [4]. However, with the rapid development of space technology and satellite imagery, large amounts of high-resolution remote sensing images are accessible now, which raised new challenges for effective remote sensing image interpretation.

Traditional segmentation methods for remote sensing images contain two steps. First, feature extractors are designed manually to mine image information, and then pixels are classified in terms of its corresponding features [5]. Several classical local features are often used, such as scale-invariant feature transform (SIFT) [6], histogram of oriented gradient HOG [7], etc. While they have been used in simple segmentation cases successfully [8,9], it is challenging to segment large-scale remote sensing images effectively due to several reasons. First, current feature extractors need to be carefully handcrafted according to the specific dataset, which have poor generalization ability on another dataset. It is unpractical to automatically analyze large amounts of remote sensing images in this way. Second, when designing a feature extractor, designers usually consider characteristics of images, such as texture, color, spectral information, spatial structure information, etc. [10,11] However, it is hard to decide which characteristic to use and how to combine several characteristics.

Recently, Convolutional Neural Networks (CNNs) [12,13] have been developing rapidly and have achieved remarkable results in many computer vision tasks. Semantic segmentation is an active topic due to its applications in remote sensing, autonomous vehicle, human activity recognition and medical image analysis, such as [14,15,16,17]. Since Fully Convolutional Networks (FCNs) [18] were proposed and successfully applied to semantic segmentation, there have been many network architectures proposed based on FCNs to improve the performance of CNNs on this task, such as Deconvolution Network [19], Deeplab [20], Spatial CNN (SCNN) [21], etc. Among the above networks, the Deeplab system has been well-known to achieve robust and efficient segmentation performance. Although these networks are successful to process common objects in daily life, such as persons, animals and vehicles, few of them concern the specific applications such as remote sensing image analysis tasks. Remote sensing images are largely different from general images from a few perspectives, such as image size, color imbalance, object rotation variation, scale variation and image distortion. In particular, CNNs cannot handle large-scale remote sensing images due to the limitation of graphics processing unit (GPU) memory.

In this work, a novel patch-wise semantic segmentation method is presented with a new training strategy to achieve fast remote sensing image analysis. To handle the large-scale image size, the image is split as many local patches according to the specific dataset at first and then a patch-wise network is developed, which specifically deals with each of the local patches. In addition, to meet the specific characteristics of remote sensing images, i.e., color imbalance, large scale variance and lens distortion, a multi-scale training strategy with special data preprocessing is proposed. The patch-wise semantic segmentation method is evaluated on a dataset that contains ten common land resources and a public dataset International Society for Photogrammetry and Remote Sensing Working Group (ISPRS WG) II/4. The experimental results show that the proposed patch-wise method and new training strategy can outperform other state-of-art methods in remote sensing image analysis. There are mainly three contributions in this work. First, a novel patch-wise semantic segmentation method is proposed to handle the large-scale image size. Second, new data augmentation is developed in several ways to handle color imbalance, rotation variations and lens distortion in remote sensing images. Third, a multi-scale training strategy is developed to handle multi-scale semantic segmentation with higher precision.

The rest of the paper is organized as follows. In Section 2, related works on remote sensing image segmentation and CNNs are reviewed. In Section 3, the proposed methods are explained in more detail. Finally, the method is evaluated on two remote sensing datasets, followed by the conclusions in Section 5.

## 2. Related Work

The study of effective segmentation methods is a hotspot in remote sensing images interpretation and there has been a lot of research on it. The common idea is to analyze remote sensing images from their pixel relations, texture structures and geographical features using methods such as region growing/merging [22], watershed algorithm [23] and support vector machine (SVM) [24]. In recent years, numerous methods have been proposed based on this idea and improved the accuracy of remote sensing image segmentation. Yang et al. [25] developed a new hybrid segmentation method that employs local spectral angle thresholds for region merging, but this method can only distinguish three landscapes with very different features. Wang et al. [26] proposed a novel image segmentation method combining superpixels with a minimum spanning tree, which obtained better image resolution and can distinguish different boundaries but think little about the difficulty of the determination of the optimal scale for different type of objects. Gaetano et al. [27] presented a new technique for the segmentation of multi-resolution remote sensing images, using an edge-based watershed to automatically process panchromatic and multispectral components.

Although lots of methods based on hand-crafted feature extractors have proven to be effective in remote sensing image analysis, there are still some problems that have not been solved very well. Firstly, generalization ability of these methods is not enough. Since remote sensing images in different periods often vary greatly due to illumination, angle of incidence and atmospheric effects, these methods often need to be adjusted based on domain expertise while transforming to new data, which hinders their ability to interpret remote sensing images with a large quantity. Second, there are many classes of objects in remote sensing images, and different classes may be very similar, such as arable land and garden. Current methods may not have enough recognition ability to distinguish multiple complex classes.

Convolution neural networks (CNNs) are a kind of mature network model in deep learning techniques, which have impressive performance in feature extraction, and have achieved remarkable results in many computer vision tasks in recent years. FCNs proposed by Long et al. [18] in 2014 is the first network to apply CNNs into semantic segmentation in the true sense. This network transformed fully connected layers into convolution layers, which enables a classification net to output a spatial map. Subsequently, many scholars have carried out a series of research studies on the application of CNNs in semantic segmentation. Noh et al. [19] presented a deep deconvolution network, using deconvolution and unpooling layers to identify detailed structures and handle objects in multiple scales, which mitigates the loss of information caused by coarse unsampling in FCNs. Lin et al. [28] formulated conditional random field (CRF) with CNN-based pairwise potential functions to capture semantic correlations between neighboring patches and applied sliding pyramid pooling to capture the patch-background context. The Deeplab system proposed by Chen et al. [20] has achieved the state-of-art results. Deeplab controls the receptive fields by an atrous convolution, and handles multi-scale objects by atrous spatial pyramid pooling (ASPP); then, the fully-connected CRF is developed as a post-process of the network.

Nevertheless, these networks were designed to process common images (usual size at around 600 to 800), and had few concerns on specific applications such as remote sensing images analysis tasks. Thus, it is problematic to directly process remote sensing images with these network models. There has been some work on transferring these common network architectures to remote sensing images. Penatti et al. [29] evaluate the generalization power of features learned by CNNs in aerial and remote sensing images. Basaeed et al. [30] proposed a fusion framework using a boosted committee of CNNs coupled with inter-band and intra-band fusion to segment multi-spectral remote sensing images. However, these two methods did not consider the large scale problem that is very important for remote sensing images. Maggiori et al. [31] constructed a fully convolutional architecture for the dense, pixelwise classification of large-scale satellite imagery and proposed a two-step training approach to address the issue of imperfect training data. However, it only achieved binary classification and cannot be applied to complex tasks. To solve these problems, this paper proposes a novel patch-wise semantic segmentation method to handle the large-scale image size. The data is preprocessed in several special ways to handle color imbalance, rotation variations and lens distortion in remote sensing images. Denoising methods [32,33] could also be used to enhance the robustness of the proposed method. Finally, a multi-scale training strategy is developed to handle multi-scale semantic segmentation with higher precision.

## 3. Methods

### 3.1. Patch-Wise Segmentation Network

**Figure 1:**
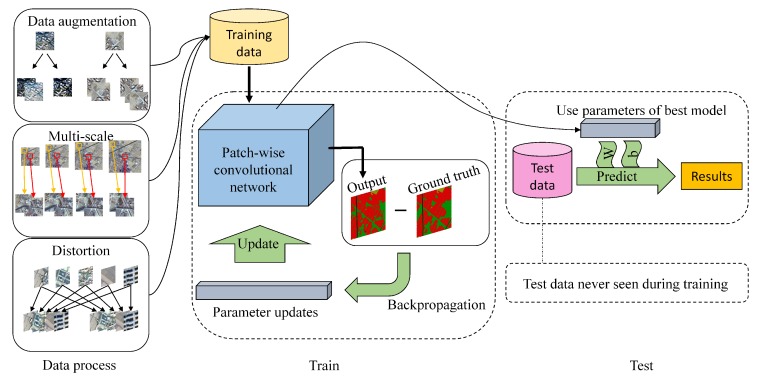
The overall architecture of the proposed method. According to the specific characteristics of high-resolution remote sensing images, this paper develops a multi-scale training strategy and new data augmentation in several ways. Then, training data is fed into the patch-wise convolutional network and parameters are updated with backpropagation. Finally, parameters of the best model are used to predict results on test data.

Remote sensing images are a special kind of image data and differ hugely from natural images, which we will talk about later. Deeplab system, a variant of FCNs, improved the performance of FCNs by modifying the structure and achieved state-of-art results in semantic segmentation tasks. There are some advantages in Deeplab that are very suitable for processing remote sensing images, so this paper takes the Deeplab system as the basic architecture to study segmentation task of remote sensing images. The overall architecture is shown in Figure 1.

First, spatial structure and texture feature are two kinds of vital information in remote sensing images. Traditional convolution strategy in FCNs and other segmentation networks lose too much of this information because of the coarse down-sampling way. To solve this problem, the Deeplab system introduced the hole algorithm [34] into CNNs and proposed a new convolution strategy—atrous convolution. Through this new convolution strategy, this paper can control the resolution of the response in a network arbitrarily and enlarge the receptive fields of filters at any CNNs layer without any extra computation. Second, the sizes of objects in remote sensing images vary widely, both inner-class and outer-class. This characteristic of remote sensing images greatly increases the difficulty of transferring segmentation networks designed for daily life images because the size of daily life objects is relatively fixed. A new structure, atrous spatial pyramid pooling (ASPP), was proposed in the Deeplab system to handle multi-scale objects without any extra parameters introduced. ASPP is the combination of atrous convolution and spatial pyramid pooling proposed in SPP-net [35]. In this structure, atrous convolutions of different atrous rates are operated on the same feature map, and outputs from these branches are fused in the end.

**Figure 2:**
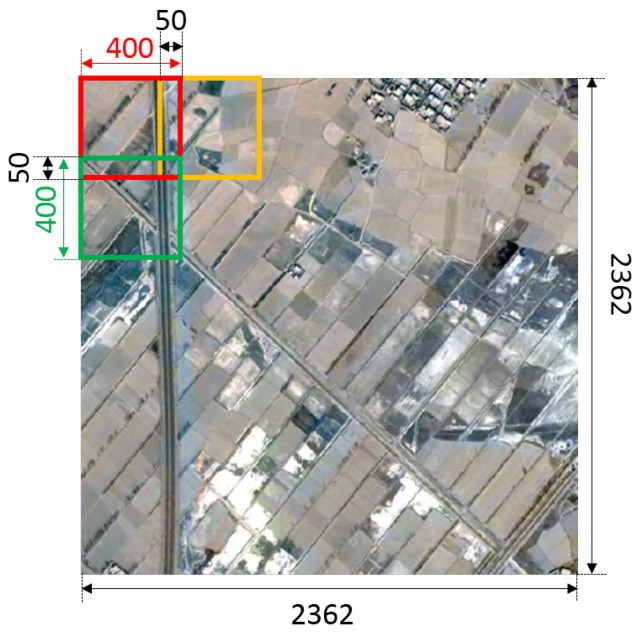
Patch-wise strategy. This paper splits the original 2362 pixels × 2362 pixels image into small patches of 400 pixels × 400 pixels with an overlapping length of 50.

**Figure 3:**
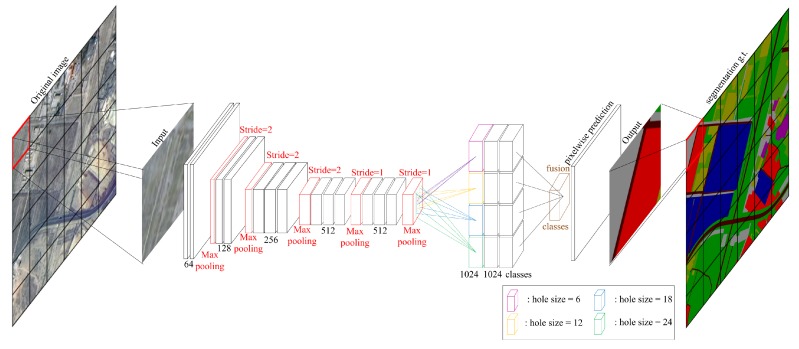
The architecture of the patch-wise convolutional network. This paper splits the large high-resolution remote sensing images into several small patches with overlap and feeds them into the deep convolutional network. Then, this paper fuses this output to get the final prediction.

However, there is still a problem in high-resolution remote sensing image analysis that Deeplab system cannot handle. Remote sensing images are usually large in size and cover a vast area, especially high-resolution remote sensing images. The Deeplab system cannot directly deal with such a large image, due to the limitation of GPU memory and other factors. Resizing images to a suitable size is a common idea, which will result in the loss of large amounts of information. This paper proposed a patch-wise strategy to deal with this problem. Firstly, the image is split into many local patches with an appropriate size according to the dataset and the GPU capacity (the size of a local patch for our dataset is 400 × 400 due to experimental results). In the process of splitting, objects in boundaries are split into two parts. Since context information is very important for CNNs to recognize objects, the split objects in boundaries will impact the performance of the patch-wise network. In order to reduce the information loss caused by splitting, a certain degree of overlapping is introduced (This paper adopts 50 pixels for the dataset after experiments). In the training process, splitting images with overlapping can increase the adequacy of our training set. More importantly, after getting the inference results on test images, adjacent images are spliced with half of the overlapping area each, so that objects in boundaries are inferred with some context information. The specific splitting method is shown in Figure 2. Then, a patch-wise network is developed to deal with each of the local patch specifically. The overall structure of the patch-wise segmentation network is shown in Figure 3. This paper evaluated the patch-wise semantic segmentation method on the dataset that contains ten common land resources. The experimental results show that the patch-wise method and new training strategy can outperform other state-of-art methods in remote sensing image analysis.

### 3.2. Special Data Preprocessing for Remote Sensing

Due to the impact of internal and external factors, such as incident angle, weather, etc., it is hard to obtain a high quality dataset containing all circumstances. Deep learning is a kind of data driven method, so it is hard to segment remote sensing images accurately under the condition of a defective dataset. This paper studied characteristics of remote sensing images and preprocessed the dataset in order to advance the performance of networks on the specific task of remote sensing image segmentation.

The color imbalance has been an intractable problem in traditional remote sensing image analysis for many years [36], and it also obstructs the performance of CNNs on this task. There are two main factors leading to this phenomenon. First, landscapes on the ground vary hugely with season changing. For instance, arable land, forest land and other objects covered by vegetation have an obvious color change over four seasons, and water may freeze in the winter. The second cause of this phenomenon lies in the imaging principle of remote sensing. Remote sensing images are synthesized according to the reflection intensity of light in each band, which is sensitive to many conditions. To start with, the incidence angle of sunlight is different in different seasons, which severely influences the reflection intensity of light. Furthermore, weather also has a serious impact on this problem. The reflection intensity of light varies hugely in different weather. In addition, the cloud may introduce extra noise to remote sensing images. In order to solve this problem, this paper augments the dataset by changing images brightness and saturation, so that networks can learn the invariance of color changes of the same object.

The problem of object rotation variations also obstructs the direct use of segmentation networks for the task of remote sensing image segmentation [37]. Natural images do not suffer from this problem too much because normal objects are usually vertical due to the Earth’s gravity. However, due to the shooting angle of remote sensing images, objects on the ground may have many different orientations, which prevents segmentation networks from extracting a common feature. In order to solve this problem, which is difficult for CNNs to handle, this paper rotated images in the dataset at three degrees—90, 180, 270. Then, this paper flipped over images in two directions—left and right, and up and down. Through these methods, the dataset was greatly augmented with objects in different orientations in order to alleviate the difficulty of using CNNs for this task and helping CNNs to extract features of different objects without orientation dependency.

**Figure 4:**
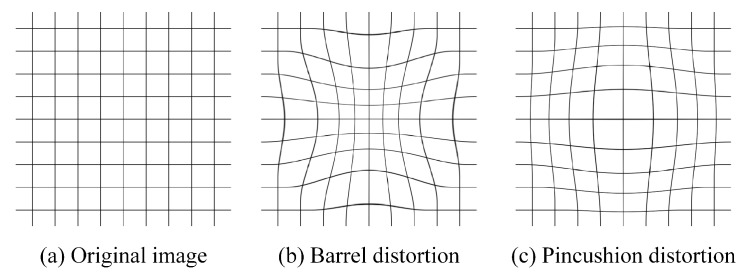
Distortion. Remote sensing images may have severe geometric distortions in the process of imaging and there are two common distortions considered in this paper.

Remote sensing is a technology that obtains information related to that observed by sensors without direct contact with them. However, due to various factors, remote sensing images may have severe geometric distortions in the process of imaging and consequently the geometry of these images does not correspond to the terrain accurately. The sources of distortion can be grouped into two broad categories: the observer and the observed. The observer refers to the acquisition system, such as satellites and sensors. Distortions caused by this factor are easy to correct according to internal parameters of the system. Factors of the second kind are more complicated and hard to control—for example, the wavy terrain, atmospheric refraction and the Earth’s rotation [38]. It is a hot research area to reduce the impact caused by second factors when interpreting remote sensing images. There are two common distortions caused by the above factors called barrel distortion and pincushion distortion [39] shown in Figure 4. CNNs are a powerful data-driven learning algorithm and their generalization ability can be improved obviously by adding additional data to the training set. In order to overcome the impact introduced by distortions when segmenting remote sensing images, this paper distorted images in the dataset in the above two ways. This paper improved the performance of network noticeably when there are distorted samples in dataset, and detailed results about distortion are shown in Section 4 separately due to the particularity of this problem.

### 3.3. Multi-Scale Training

Remote sensing images are special kinds of images that differ hugely from natural images. One of the main differences is that the scale of objects in remote sensing images varies widely while it is relatively fixed in natural images. The wide scale variation of common land resource objects in remote sensing images is mainly reflected in two aspects. Firstly, there are significant differences in the scale between objects of different categories. For instance, objects in categories like arable land and forest land usually cover a wide area and distribute in a patchy way while objects in categories like road and river are usually thin and linear. Thus, it is extremely difficult to design a classifier that can accurately segment large objects while taking small objects into account. Secondly, there might be significant differences in the scale between objects of the same category as well. For instance, metropolitan buildings are usually concentrated in large areas while buildings in the countryside are usually fragmentarily scattered around the arable land and forest land. This point makes it difficult to effectively capture invariable features of objects and affect the accuracy of networks.

The scale variation problem is an important factor that limits the results of using traditional segmentation methods to process remote sensing images. It is also an important reason why this paper cannot use segmentation networks designed for general tasks to process remote sensing images directly. Hence, a model is required that is sensitive enough to the scale so that we can distinguish objects in different categories but also robust enough so that we can identify objects in the same category that may have different scales. There are some common ways to enhance the ability of network to handle multi-scale objects. Considering objects of different scales in the process of network designing can improve the performance to handle multi-scale objects—for example, the Deeplab system using ASPP structure to handle multi-scale objects. However, it still cannot meet the requirement to deal with the wide-scale variation problem in semantic segmentation tasks. By training CNNs with a dataset containing images of different scales, it can further improve their ability to handle multi-scale objects, in order to obtain a model with the ability to abstract features of objects in different scales [40]. As for semantic segmentation tasks, Chen et al. [41] demonstrated that training with multi-scale images can get a better segmentation result than single-scale images in three datasets—PASCAL-Person-Part, PASCAL VOC 2012 and MS-COCO 2014. Therefore, a strategy is proposed to train networks with multi-scale images to solve the severe scale variation problem for the specific task of remote sensing image semantic segmentation, and it has achieved great results.

The raw data is resized and gets images of four scales in total: 1.25, 1, 0.75, and 0.5. Remote sensing images are very large in general, so, after resizing, this paper cropped images to 400 × 400, a universal size used in segmentation networks that has great tradeoff between memory requirements and network performance. Training a network with a dataset that incorporates images of these four scales can greatly improve the generalization ability of the network, help the network to get rid of the scale dependency when handling remote sensing images and learn the invariable features of the objects in each category. Experiments showed that this multi-scale training strategy can effectively solve the wide scale difference in remote sensing images and greatly improve the performance of deep learning network models in the task of remote sensing image semantic segmentation.

## 4. Experimental Results

### 4.1. Setup

**Figure 5:**
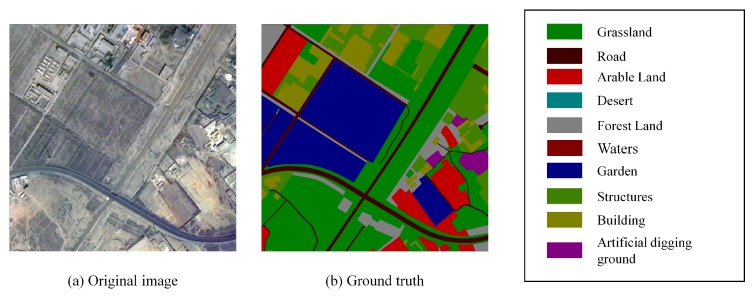
An example from the dataset. A remote sensing image and its ground truth, alongside the class legend.

***Dataset*** The proposed strategy is evaluated on an image set of 341 high-resolution images collected from a capital city in West China in the year 2016 with the Gaofen-2 satellite. The average image size is 2362 × 2362 pixels (approximately 1.411 billion pixels in total). The spatial resolution of the images is 2 m/pixel so the image set covers an area of about 8000 square kilometers. The data is manually annotated into 10 common land resources (Grassland, Road, Building, Arable Land, Structures, Desert, Forest Land, Artificial digging ground, Waters, Garden) at the pixel-level by visual interpretation and then the annotations were corrected by field investigation. This is a huge amount of work and took a few months. This work is the data basis of the research in this paper on remote sensing image segmentation tasks. Specifically, the image set consists of 16,710 images in total after splitting to 400 × 400 patches. In addition, 11,809 images are used to train the model and the rest is used to evaluate the performance. An example patch of a remote sensing image along with the class legend from the dataset is displayed in Figure 5 alongside the corresponding ground truth image.

***Evaluation metrics*** The evaluation metrics for the proposed strategy are inspired by FCNs. There are four different criteria: pixel accuracy (*PA*), mean accuracy (*MPA*), mean intersection over union (*MIoU*), frequency weighted intersection over union (*FWIoU*).

Let $n_{ij}$ be the number of pixels of class *i* predicted to belong to class *j*, where there are $n_{cl}$ different classes, so $n_{ii}$ is the number of pixels of class *i* predicted correctly. This paper computes:

(1) $$\begin{array}{c} PA=\sum_{i=0}^{n_{cl}}n_{ii}/\sum_{i=0}^{n_{cl}}\sum_{j=0}^{n_{cl}}n_{ij},\\ MPA=\left(1/n_{cl}\right)\sum_{i=0}^{n_{cl}}n_{ii}/\sum_{i=0}^{n_{cl}}\sum_{j=0}^{n_{cl}}n_{ij},\\ MIoU=\left(1/n_{cl}\right)\sum_{i=0}^{n_{cl}}n_{ii}/\left(\sum_{j=0}^{n_{cl}}n_{ij}+\sum_{j=0}^{n_{cl}}n_{ji}-n_{ii}\right),\\ FWIoU={\left(\sum_{i=0}^{n_{cl}}\sum_{j=0}^{n_{cl}}n_{ij}\right)}^{-1}\sum_{i=0}^{n_{cl}}\left(\sum_{j=0}^{n_{cl}}n_{ij}n_{ii}\right)/\left(\sum_{j=0}^{n_{cl}}n_{ij}+\sum_{j=0}^{n_{cl}}n_{ji}-n_{ii}\right). \end{array}$$

In short, *PA* is the ratio of pixels classified correctly to the total pixels and *MPA* is the average of the per-class average accuracies. *MIoU* is a standard measure metric that represents the ratio of intersection to union between the prediction and ground truth. *FWIoU* is an improved over the raw *MIoU* that weights each class importance depending on their appearance frequency.

In the following section, the experiment and comparison will be presented to evaluate the approach. All experiments in this paper were performed using the deep learning framework Caffe [42]. The network is based on the publicly available model, Deeplabv2 VGG16, which advances the results on the PASCAL VOC-2012 semantic image segmentation task. This paper follows the methodology of [13] in training the datasets, in terms of learning rate, momentum parameter selection and weight decay, setting a mini-batch size of 15 images and initial learning rate of 0.001. The learning rate is multiplied by 0.1 after 2000 iterations, using weight decay of 0.0005 and the momentum of 0.9. At the same time, the network utilizes the weights of the VGG16 pre-trained model on ImageNet as the initialization. The training process took 20 h on four NVIDIA GTX 1080Ti GPUs and the inference time for each image in the test dataset is about 0.12 s on a single GTX 1080Ti.

### 4.2. Patch-Wise Configuration

**Figure 6:**
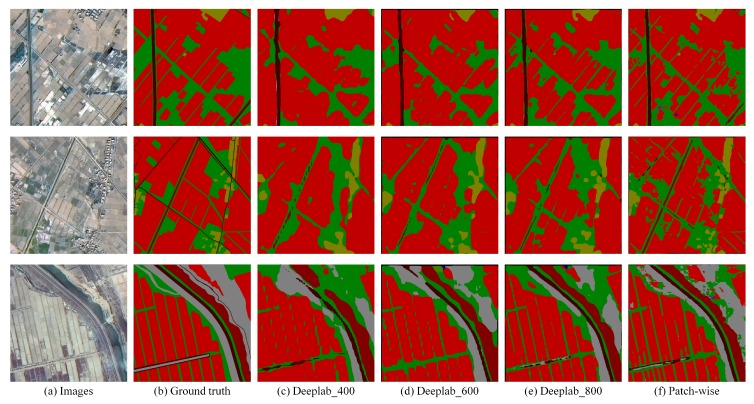
Comparison of the patch-wise method and Deeplab with different resize scales. This method shows superior performance, particularly with its ability to correctly segment and delineate boundaries, as compared to Deeplab with different resize scales (400, 600, 800). The most obvious improvement occurring due to this method is the segmentation of roads.

In this section, the results of the patch-wise strategy and the resize strategy with different scales are compared. Remote sensing images are usually large in size and cover a vast area. However, due to GPU memory and other factors, the Deeplab system cannot directly deal with such a large image. Resizing images to a suitable size is a common idea, which will result in the loss of large amounts of information. A strategy is proposed to deal with this problem by splitting the image into many small local patches and then developing a patch-wise network to deal with each of the local patch specifically. This paper splits the large image in the dataset to a size of 400 × 400 with an overlapping length of 50. (Different crop size can also affect the performance of the algorithm. Large crop size makes the output smoother while small crop size makes the details more precise. Several different crop sizes are tested and 400 × 400 is most suitable for our dataset, which balances macroscopic effects and detail accuracy.) Through this strategy, the accuracy is greatly improved for remote sensing image segmentation and the details are shown in Table 1. As shown in Table 1, all four indexes have been promoted, and the improvement of MPA and MIoU is astonishing. This is because most of the area covered by remote sensing images is arable land, which is usually continuous and vast. The resize operation does not cause too much loss of information on these targets, but seriously damages the details of small targets such as roads and rivers. Therefore, the improvement in overall accuracy is small but gets obvious while taking into account the factor of ten categories. Another piece of evidence regarding the information loss phenomenon of the resize operation is that accuracies are gradually increased as the resize scale increases. This paper visualizes the effect of the different strategies in Figure 6.

### 4.3. Data Preprocessing and Multi-Scale Training

***Data preprocessing*** The color of remote sensing images is severely influenced by several factors. In order to solve this problem, the dataset is augmented in color by changing image intensity and saturation, so that networks can learn the invariance of color changes of the same object. The brightness of each image is increased/decreased by 50% and decreases its contrast so that this research gets a 3× data expansion. At the same time, the problem of object rotation variations also obstructs the segmentation of remote sensing images. The images are rotated in the dataset at three degrees—90, 180, 270—and flipped over in two directions—left and right, and up and down. Through this operation, the data is expanded in 5× and the diversity of the dataset is increased.

***Multi-scale training*** A wide range of variation of common land resource objects in remote sensing images exists. By training CNNs with a dataset containing images of different sizes, it can further improve their ability to handle multi-scale objects. This paper resized the raw data and obtained images of four scales in total: 1.25, 1, 0.75, and 0.5. Then, the images were cropped to 400 × 400, a universal size used in segmentation networks that has great trade-off between memory requirement and network performance.

**Figure 7:**
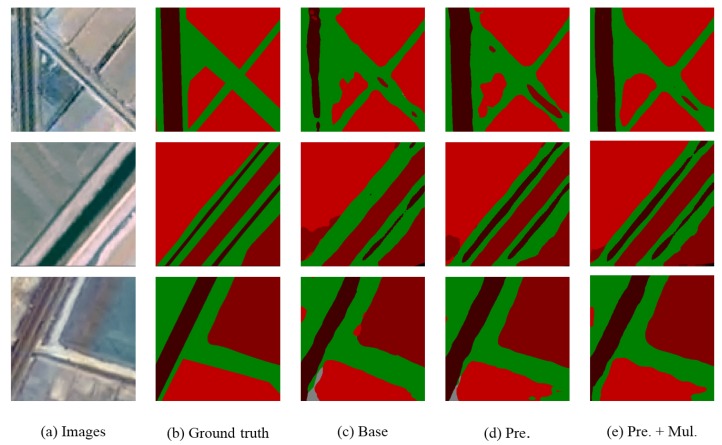
Segmentation results of remote sensing. (**a**) input image; (**b**) ground truth; (**c**) train a model with the original training set and without any data preprocessing; (**d**) train a model with the training set after data preprocessing and (**e**) add multi-scale data into the preprocessed dataset.

As shown in Figure 7, through data preprocessing and multi-scale operation, the segmentation results of remote sensing images are greatly improved: (1) segmentation boundaries are more accurate; (2) the ability to capture small objects is more powerful; and (3) there are less objects that are misclassified. The detailed accuracies are shown in Table 2.

**Figure 8:**
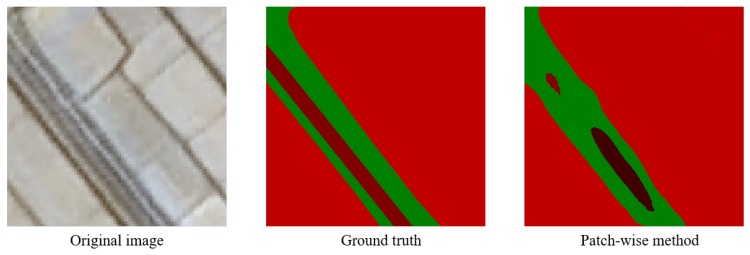
While dealing with spindling objects such as roads, the network sometimes fails to achieve accurate segmentation results.

***Shortcomings*** The proposed patch-wise method achieves good results in the segmentation of remote sensing images, but there are still some shortcomings. The method works well for blocky, contiguous targets such as arable land, desert and water. As for spindling objects such as road, the network sometimes fails to recognize. The boundary details of the segmentation results also need to be improved. Figure 8 is an example of not segmenting the road accurately.

### 4.4. Distortion

Image distortion is an intractable problem in the interpretation of remote sensing images. By being trained with a dataset containing distortion samples, a CNN model may get the ability to resist the impact brought by image distortion. The images are manually distorted in the dataset in two ways according to the causes of distortions: barrel distortion and pincushion distortion. As shown in Table 3, while testing with a dataset containing distortion samples, all four evaluation metrics dropped obviously compared with Table 2—about 10% for PA and FWIoU, 25% for MPA and MIoU. The reason why the MPA and MIoU dropped much more is that the impact of distortion phenomenon is greater on small targets. By training with a dataset containing distortion samples, the accuracy of remote sensing image segmentation is improved with considerable percentage points for each metric while a distortion phenomenon exits.

### 4.5. Impact of CRF

Chen et al. [20] improved the performance of Deeplab by using fully connected CRF to capture the delicate boundaries of objects. This paper has also studied the effect of CRF on remote sensing image segmentation.

**Figure 9:**
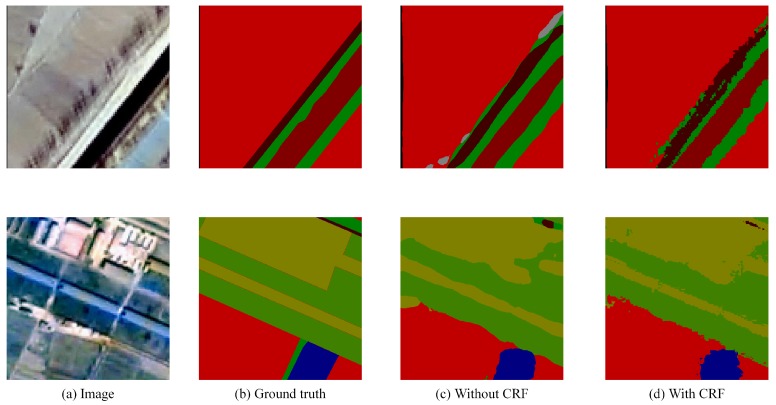
Better results with CRF. Post-processing with CRF makes segmentation boundaries of objects more clear and improves the overall accuracy of remote sensing image segmentation.

**Figure 10:**
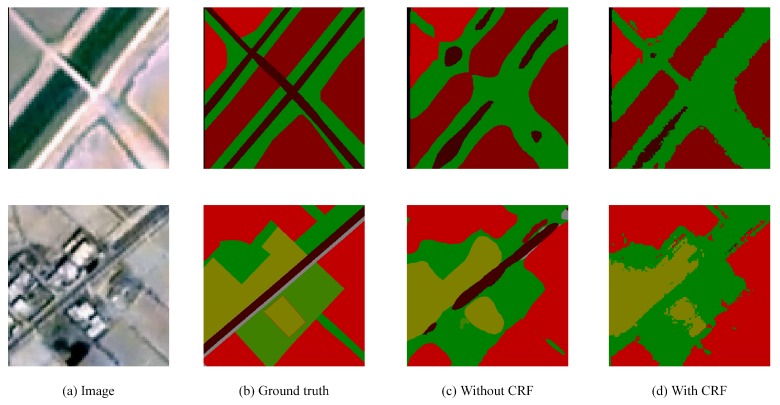
Failure results with CRF. While processing with CRF, there are some samples where the improvement is not obvious or even worse, especially for small objects such as roads, which may disappear after processing.

In general, like other tasks, postprocessing with CRF makes boundaries of objects more clear and improves the overall accuracy of remote sensing image segmentation. However, there are also some samples where the improvement is not obvious or even worse, especially for objects such as roads. It is most likely that remote sensing images are more blurred than ordinary natural images, and the pixels of them are arranged in a grid. Therefore, post-process remote sensing images with CRF may excavate the relationship of each pixel and the details of images excessively, which results in a worse result of the overall segmentation. The detailed results are shown in Figure 9 and Figure 10.

**Figure 11:**
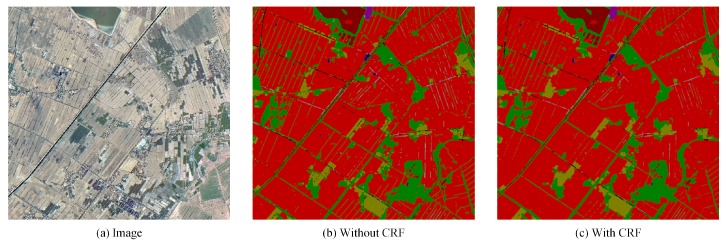
Results on a capital city in West China. This paper tests a remote sensing image that covers 20 square kilometers in a capital city of China and the patch-wise network shows brilliant performance. Post-processing with CRF can reduce the impact of noise and make segmentation results more continuous.

At the same time, this paper tested a remote sensing image that covers 20 square kilometers in a capital city in West China and achieves great performance, as shown in Figure 11. In this sample, post-processing with CRF reduces the impact of noise and makes segmentation result more continuous.

### 4.6. Evaluation on Public Dataset

**Figure 12:**
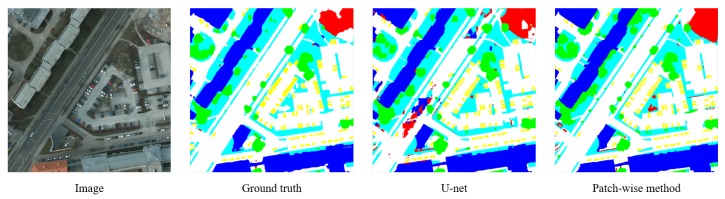
Segmentation results of U-net and the patch-wise method. The patch-wise method can achieve more accurate segmentation results with less misidentified objects.

The proposed patch-wise method is also evaluated on a public state-of-the-art airborne image dataset, ISPRS WG II/4, and is compared to other baseline methods. Images of this dataset taken from Potsdam show a typical historic city with large building blocks, narrow streets and a dense settlement structure. The dataset has been classified manually into the six most common land cover classes (Impervious surfaces, Building, Low vegetation, Tree, Car, Clutter/background). Table 4 is the comparison between the patch-wise method and three state-of-the-art methods according to pixel accuracy. Figure 12 is an example of the segmentation results of the patch-wise method and U-net.

## 5. Conclusions

This paper has presented a novel patch-wise semantic segmentation method for high-resolution remote sensing images. First, high-resolution remote sensing images are split into many small local patches and then developed a patch-wise network. Second, according to the specific characteristics of remote sensing images, the data was preprocessed in several ways to improve the robustness of the proposed model for remote sensing images. Third, a multi-scale training strategy was developed to solve the severe scale variation problem in remote sensing images. The patch-wise semantic segmentation method was evaluated on ISPRS WG II/4 and a dataset was collected from a capital city in West China, and outperformed other state-of-art methods.

## Figures and Tables

**Table 1:** Comparison of the patch-wise semantic segmentation method and Deeplab with different resize scales (400, 600, 800). The bold numbers mean that they are the best results.

	PA	MPA	MIoU	FWIoU
Deeplab_400	0.7401	0.3294	0.2514	0.6185
Deeplab_600	0.7508	0.4402	0.2714	0.6259
Deeplab_800	0.7612	0.4684	0.2995	0.6515
Patch-wise	**0.7877**	**0.5978**	**0.4482**	**0.6904**

**Table 2:** Through data preprocessing and multi-scale training, the accuracy of remote sensing image segmentation is improved. The bold numbers mean that they are the best results.

	PA	MPA	MIoU	FWIoU
Base	0.7877	0.5978	0.4482	0.6904
Pre.	0.8218	0.6425	0.4984	0.7214
Pre. + Mul.	**0.8521**	**0.6723**	**0.5496**	**0.7619**

**Table 3:** Training with a dataset containing distortion samples, the accuracy of remote sensing image segmentation is improved while a distortion phenomenon exits. The bold numbers mean that they are the best results.

	PA	MPA	MIoU	FWIoU
Base	0.7404	0.4148	0.3011	0.6585
Pre.	0.7444	0.4209	0.3057	0.6622
Pre. + Mul.	0.7459	0.4235	0.3138	0.6704
Distortion	**0.7828**	**0.4674**	**0.3500**	**0.7218**

**Table 4:** Comparison with three state-of-the-art methods. The proposed patch-wise method outperforms them according to PA. SVL_1 is based on an Adaboost-based classifier and CRF, U-net and CNN-FPL are both based on CNNs. The bold number means that it is the best result.

Method	SVL_1 [43]	U-net [44]	CNN-FPL [45]	Patch-Wise Method
PA	0.778	0.832	0.858	**0.861**
